# Supplementary figures and images for: Temperature cues are integrated in a flexible circadian neuropeptidergic feedback circuit to remodel sleep-wake patterns in flies
Source: PLoS Biol. 2024 Dec 2;22(12):e3002918. doi: 10.1371/journal.pbio.3002918 (PMC11611155; doi:10.1371/journal.pbio.3002918)

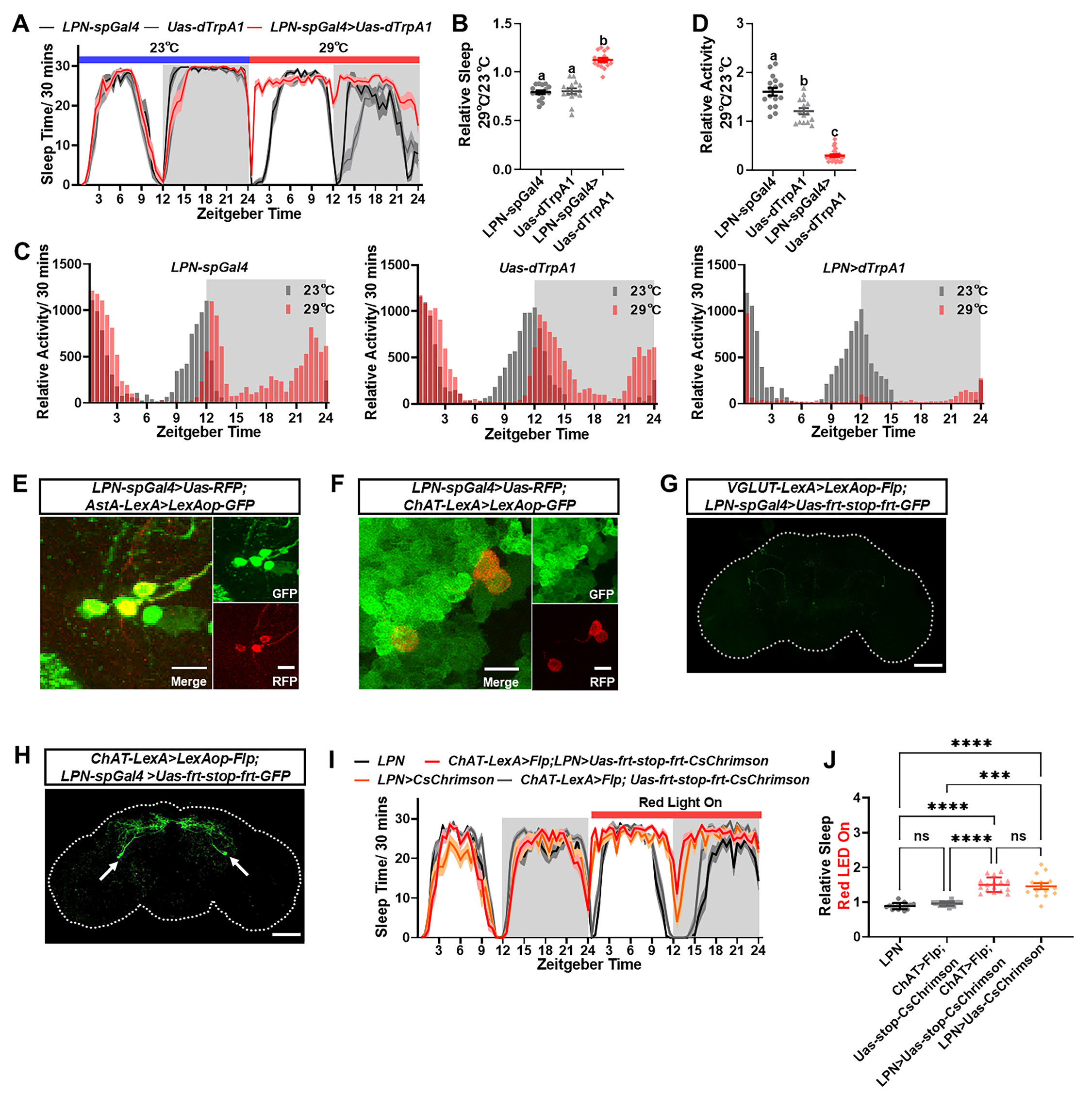

Supplement: S1 Fig — (A, B) LPN activation via the thermogenetic dTrpA1 channel (LPN-spGal4>Uas-dTrpA1; red line) significantly increases sleep compared to baseline. (C, D) LPN activation via the thermogenetic dTrpA1 channel (LPN-spGal4>Uas-dTrpA1) significantly decreases daily locomotion compared to baseline (black bar). (E) AstA neurons (AstA-LexA>LexAop-GFP; green) are colocalized with LPNs (LPN-spGal4>Uas-RFP; red). Scale bar, 10 μm. (F) ChAT neurons (ChAT-LexA>LexAop-GFP; green) are colocalized with LPNs (LPN-spGal4>Uas-RFP; red). Scale bar, 10 μm. (G, H) Intersection neurons between VGLUT-LexA (G) or ChAT-LexA (H) and LPN-spGal4 are labeled with GFP. Intersection with ChAT-LexA clearly marks LPNs (arrows). Scale bar, 100 μm. (I, J) Activation of intersection-labeled LPNs (red line) has a sleep-promoting effect similar to LPN-spGal4 activation (yellow line). All data were analyzed using Welch’s one-way ANOVA with multiple comparisons, and letters a, b, and c indicate significant differences, P < 0.05. ns = not significant, ***P < 0.001, ****P < 0.0001. The raw data in this figure including A, B, C, D, I, and J can be found in S1 Data. (TIF) [file pbio.3002918.s004.tif]

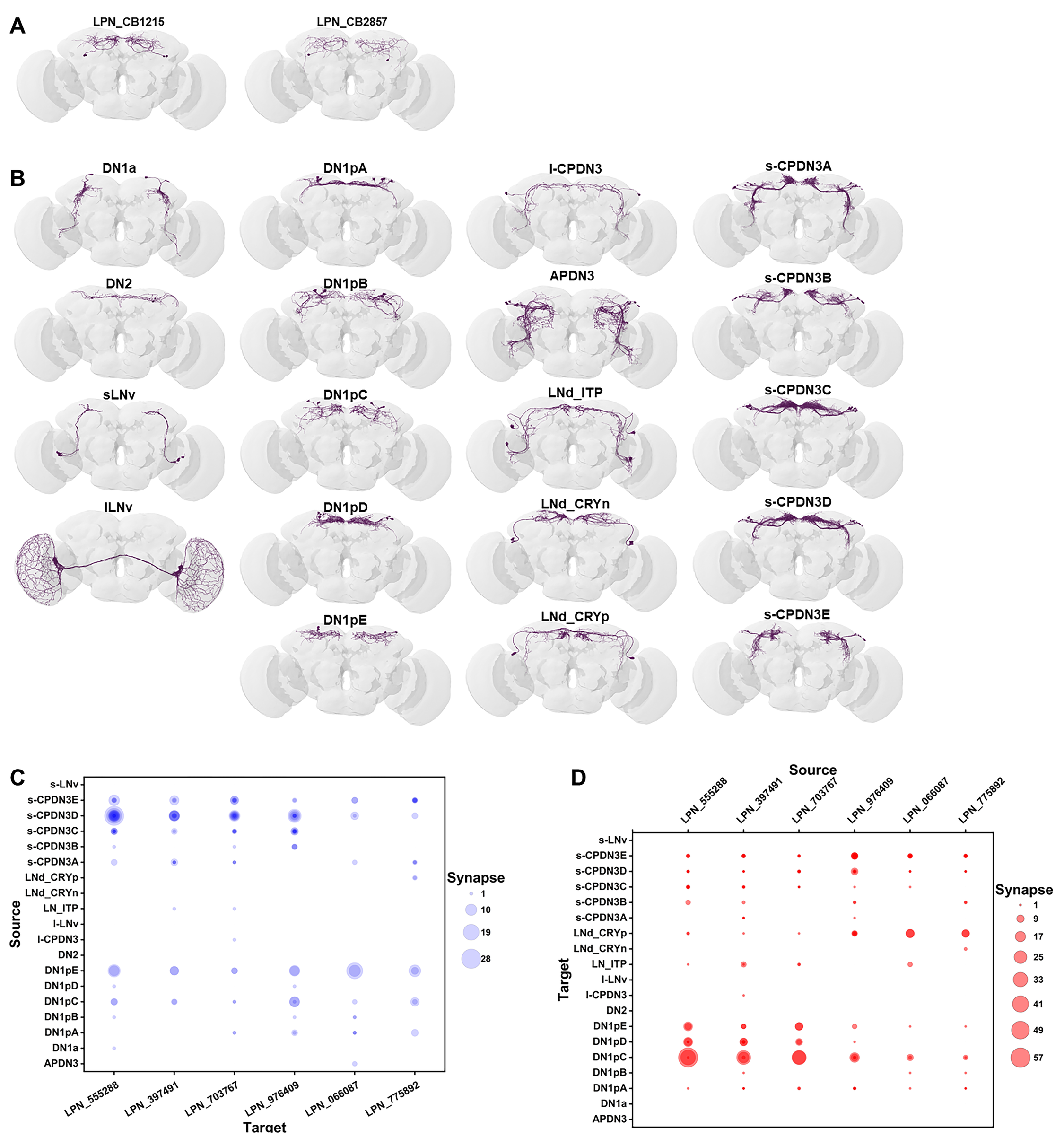

Supplement: S2 Fig — (A) EM reconstruction of LPN subtypes CB1215 and CB2857 in Flywire database. (B) EM reconstruction of recently annotated circadian cell types in Flywire database. (C, D) Quantification of presynaptic inputs to LPNs from all annotated circadian neurons (C) and postsynaptic outputs from LPNs to all annotated circadian neurons (D). Circle size represents the number of synapses and each circle means one single neuron. (TIF) [file pbio.3002918.s005.tif]

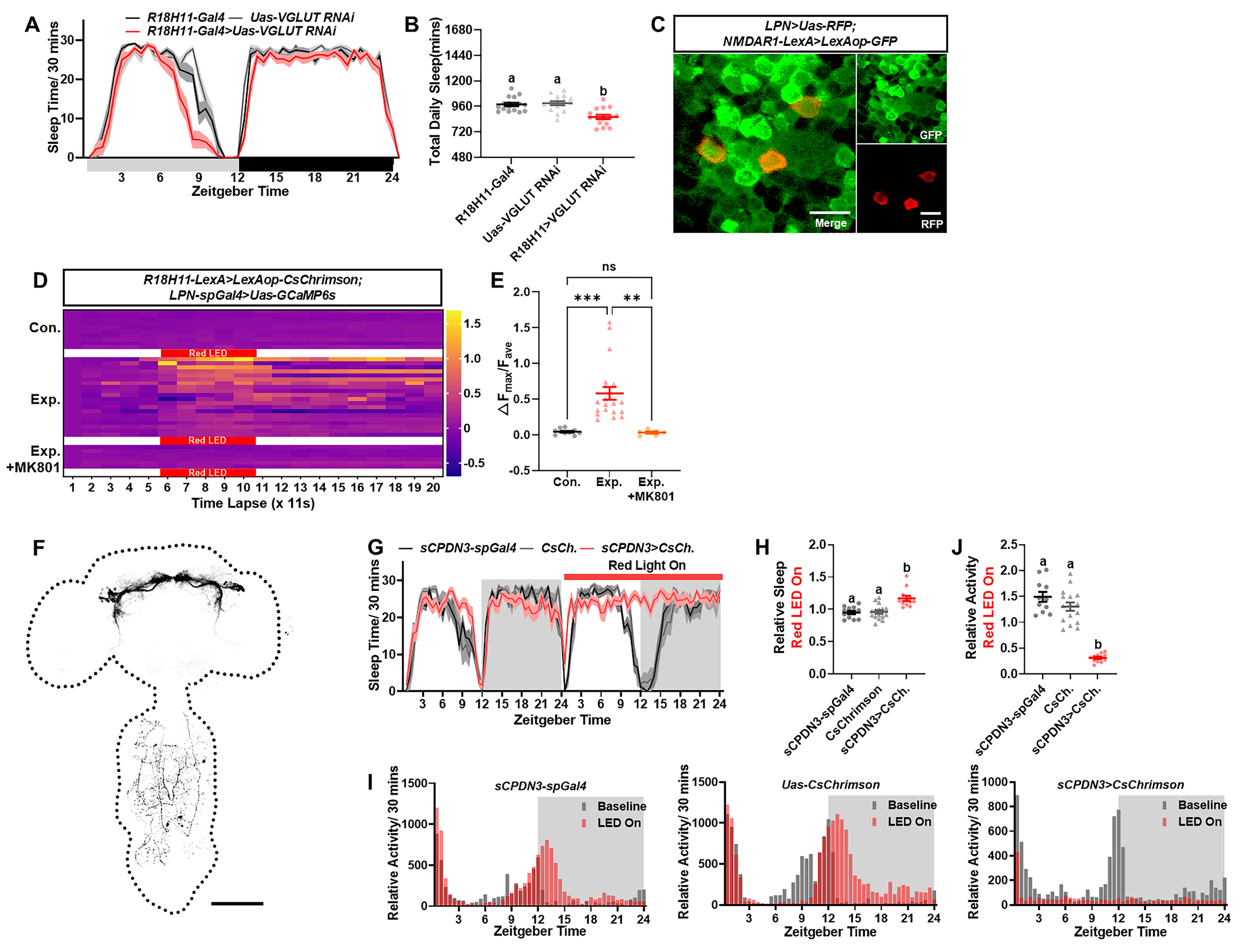

Supplement: S3 Fig — (A, B) VGLUT knockdown in DN1ps (R18H11-Gal4>Uas-VGLUT RNAi) reduces daily sleep duration. Sleep patterns (A) and total sleep time (B) are shown. (C) NMDAR1 neurons (NMDAR1-LexA>LexAop-GFP; green) are colocalized with LPNs (LPN-spGal4>Uas-RFP; red). Scale bar, 10 μm. (D, E) Optogenetic activation of DN1ps (R18H11-LexA>LexAop-CsChrimson) increases LPN calcium levels in vivo, and Glu antagonist-MK801 blocks this activation. Heat maps (D) and normalized maximum calcium levels (E) are shown. Z-stacks of 10 frames with 1.1 s intervals. (F) Confocal images of sCPDN3-spGal4 (sCPDN3-spGal4>Uas-GFP) showing brain and ventral nerve cord. Scale bar, 100 μm. (G, H) Optogenetic activation of sCPDN3 (sCPDN3-spGal4>Uas-CsChrimson; red line) significantly increases sleep compared to baseline. (I, J) Optogenetic activation of sCPDN3 (sCPDN3-spGal4>Uas-CsChrimson) significantly decreases daily locomotion compared to baseline (black bar). Data (E) were analyzed with an unpaired t test. Data (B, H, J) were analyzed using Welch’s one-way ANOVA with multiple comparisons, and letters a and b denote significant differences, P < 0.05. The raw data in this figure including A, B, D, E, G, H, I, and J can be found in S1 Data. (TIF) [file pbio.3002918.s006.tif]

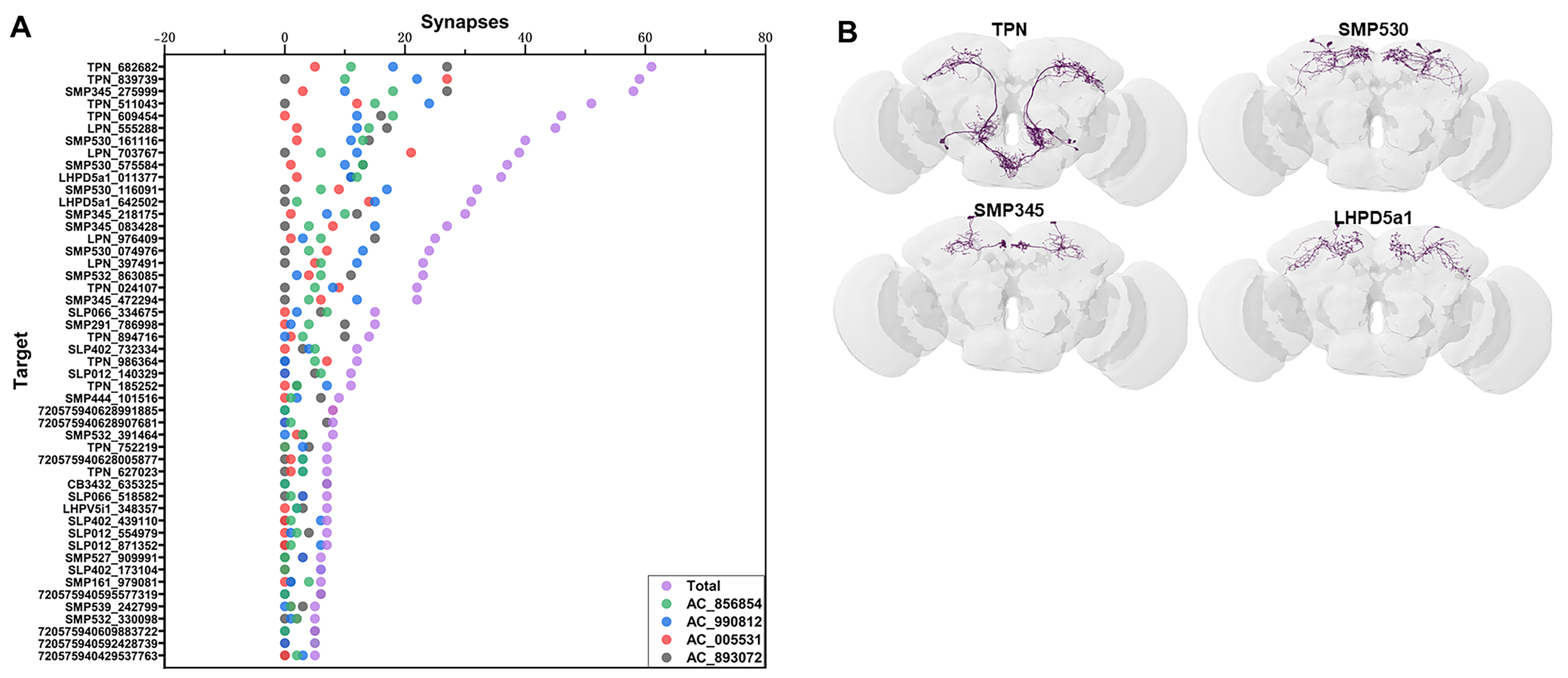

Supplement: S4 Fig — (A) Top 50 postsynaptic neurons of ACs from the FlyWire Data set (v783). (B) EM reconstruction of AC postsynaptic neurons from panel A. (TIF) [file pbio.3002918.s007.tif]

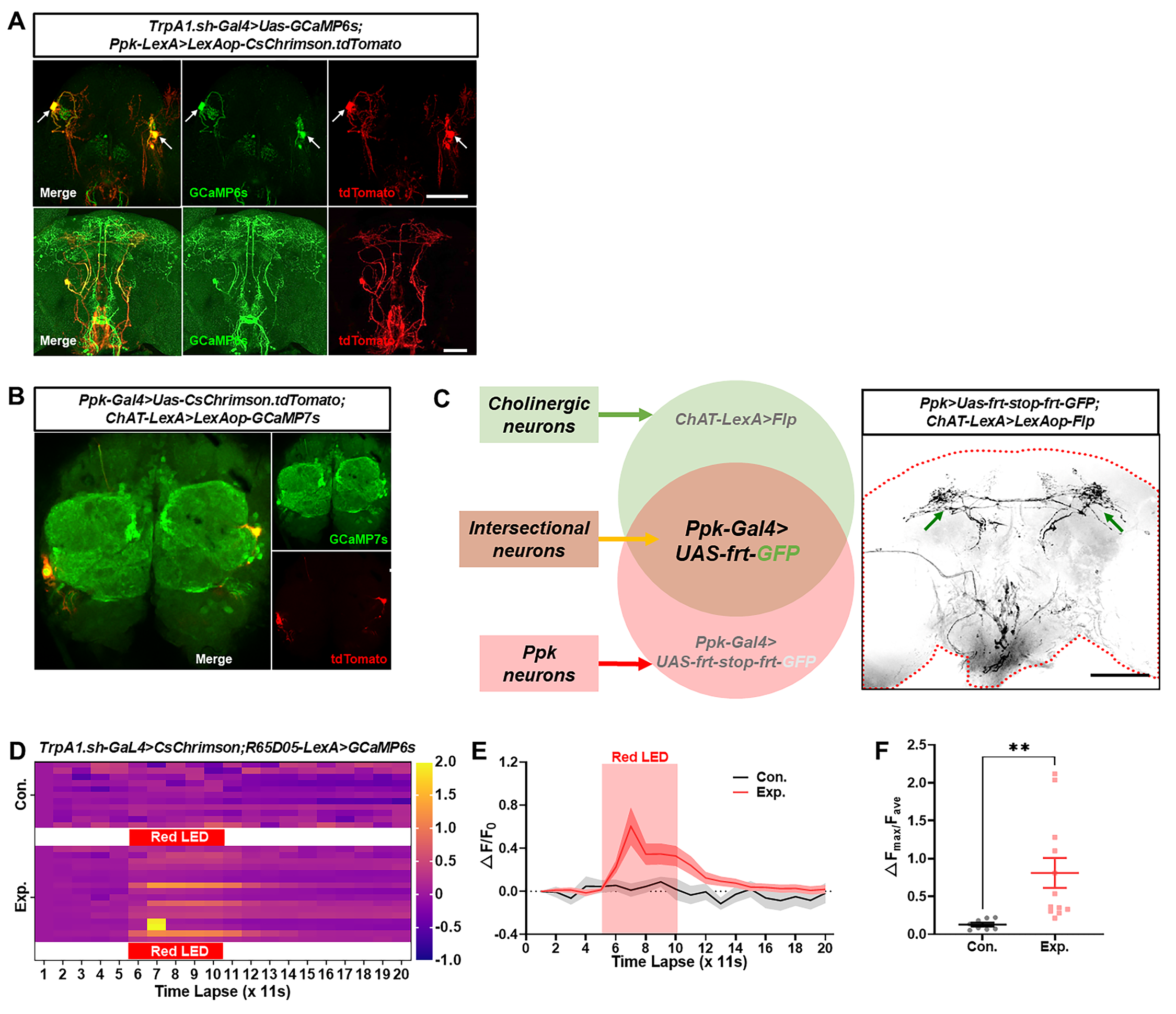

Supplement: S5 Fig — (A) Ppk neurons (Ppk-LexA>LexAop-CsChrimson.tdTomato; red) are colocalized with TrpA1.sh neurons (TrpA1.sh-Gal4>Uas-GCaMP6s; green). Scale bar, 50 μm. (B, C) ACs are cholinergic. ChAT neurons (ChAT-LexA>LexAop- GCaMP7s; green) are colocalized with Ppk neurons (Ppk-Gal4>Uas- CsChrimson.tdTomato; red) (B). AC neurons are clearly labeled by intersection between ChAT-LexA and Ppk-Gal4 (arrows) (C). Scale bar, 100 μm. (D–F) Optogenetic activation of TrpA1.sh neurons with CsChrimson (TrpA1.sh-Gal4>Uas-CsChrimson) promotes LPN calcium levels in vivo. Heat maps (D), a summarized line graph (E) and normalized calcium levels (F) are shown. Z-stacks of 10 frames with 1.1 s intervals. Unpaired t test. **P < 0.01. The raw data in this figure including D, E, and F can be found in S1 Data. (TIF) [file pbio.3002918.s008.tif]

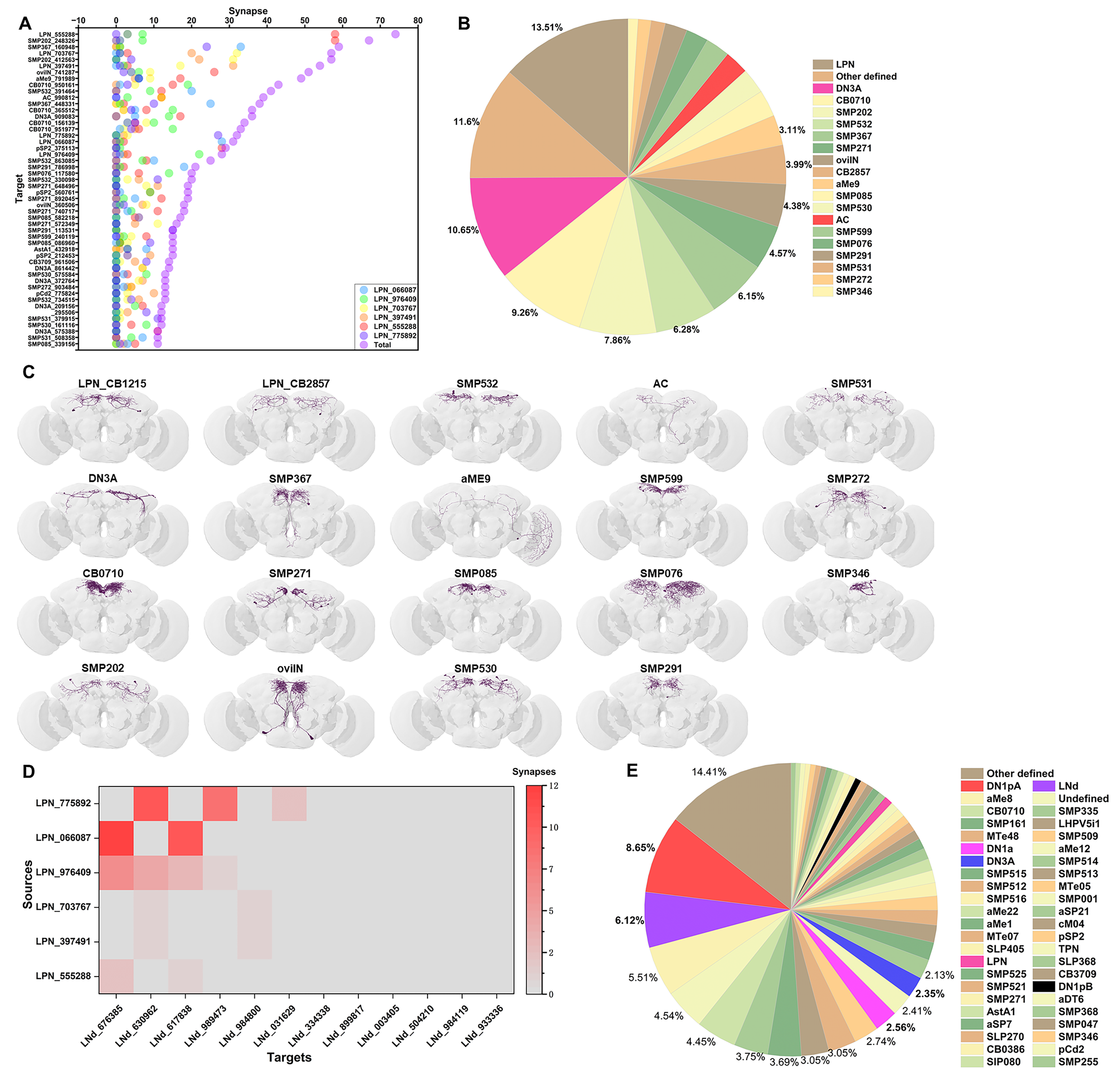

Supplement: S6 Fig — (A) Top 50 postsynaptic neurons of LPNs based on FlyWire Data set (v783). (B) Percentage of output from LPNs to various neuron types with ≥5 synapses, as predicted using Flywire data set. (C) EM reconstruction of LPN postsynaptic neurons from panel B. (D) Synaptic connections from each LPN to each LNd. (E) Percentage of input to LNds from different neuron types with ≥5 synapses, as predicted using Flywire data set. (TIF) [file pbio.3002918.s009.tif]

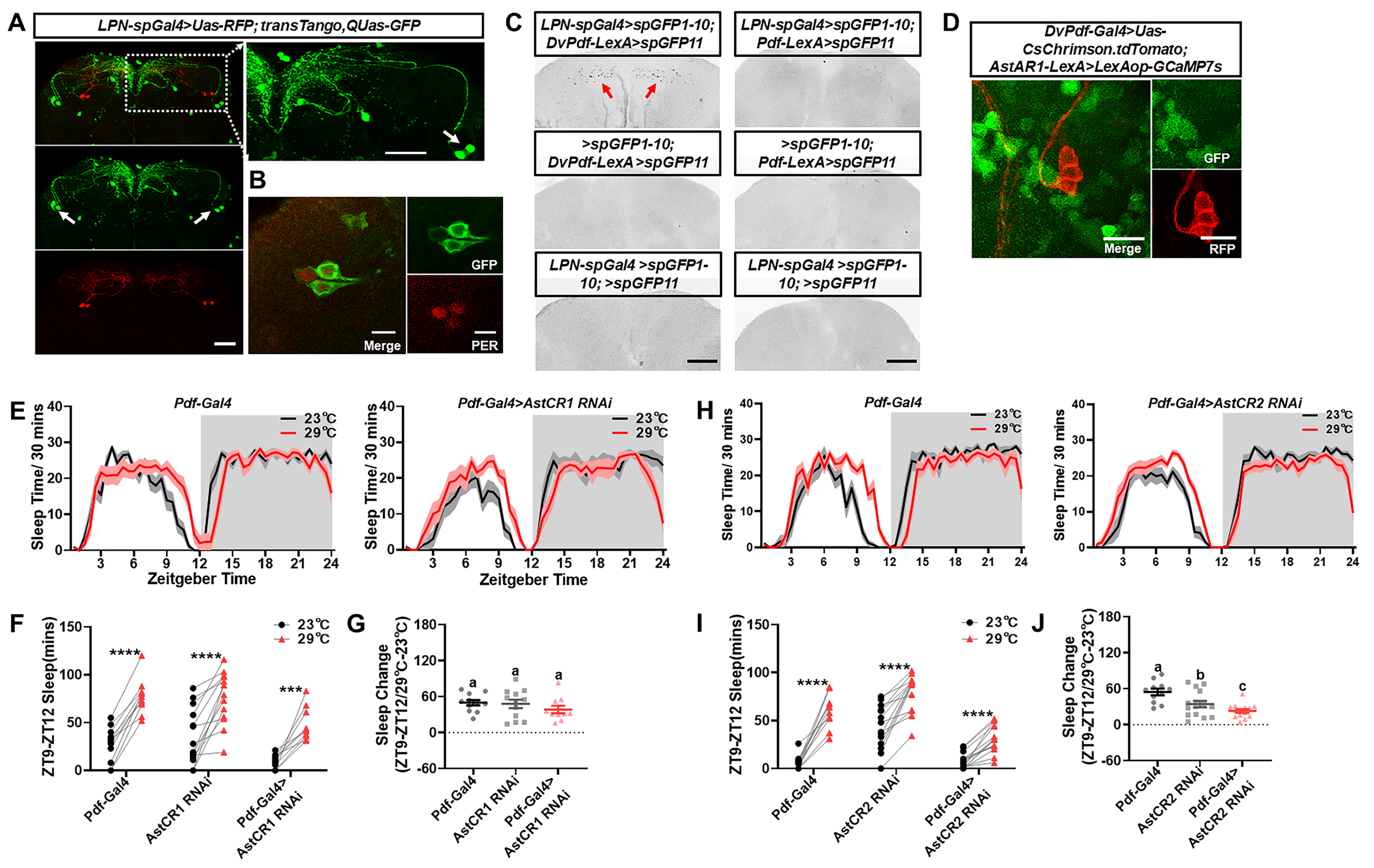

Supplement: S7 Fig — (A) LPNs (red) and their postsynaptic targets (green) shown with LPN-spGal4>Uas-RFP; transTango,QUas-GFP. Arrows indicate LNd-like cells. Scale bar, 50 μm. (B) Co-staining of PER (red) with all LPN downstream targets (LPN-spGal4>transTango,Quas-GFP; green), highlighting the circadian neuron identity. Scale bar, 10 μm. (C) LPNs form synaptic spatial overlaps with LNds (LPN-spGal4>Uas-sp1-10;DvPdf-LexA>LexAop-GFP11; left) but not with sLNv (LPN-spGal4>Uas-sp1-10;Pdf-LexA>LexAop-GFP11; right). Scale bar, 50 μm. (D) AstAR1 neurons (AstAR1-LexA>LexAop-GCaMP7s; green) did not colocalized with LNds (DvPdf-Gal4>Uas-CsChrimson.tdTomato; red). Scale bar, 10 μm. (E–G) Evening sleep increase (ZT9-ZT12) at high temperature (29°C, red line) compared to baseline (23°C, black line) was unaffected by AstCR1 knockdown in LNvs (Pdf-Gal4>Uas-AstCR1 RNAi). Quantification of evening sleep (F) and sleep change (G) before and after temperature increase. (H–J) Evening sleep increase (ZT9-ZT12) at high temperature (29°C, red line) compared to baseline (23°C, black line) was blocked by AstCR2 knockdown in LNvs (Pdf-Gal4>Uas-AstCR2 RNAi). Quantification of evening sleep (I) and sleep change (J) before and after temperature increase. Data (G, J) were analyzed using Welch’s one-way ANOVA with multiple comparisons; letters a, b, and c denote significant differences, P < 0.05. Data (F, I) were analyzed with paired t test. ***P < 0.001, ****P < 0.0001. The raw data in this figure including E, F, G, H, I, and J can be found in S1 Data. (TIF) [file pbio.3002918.s010.tif]

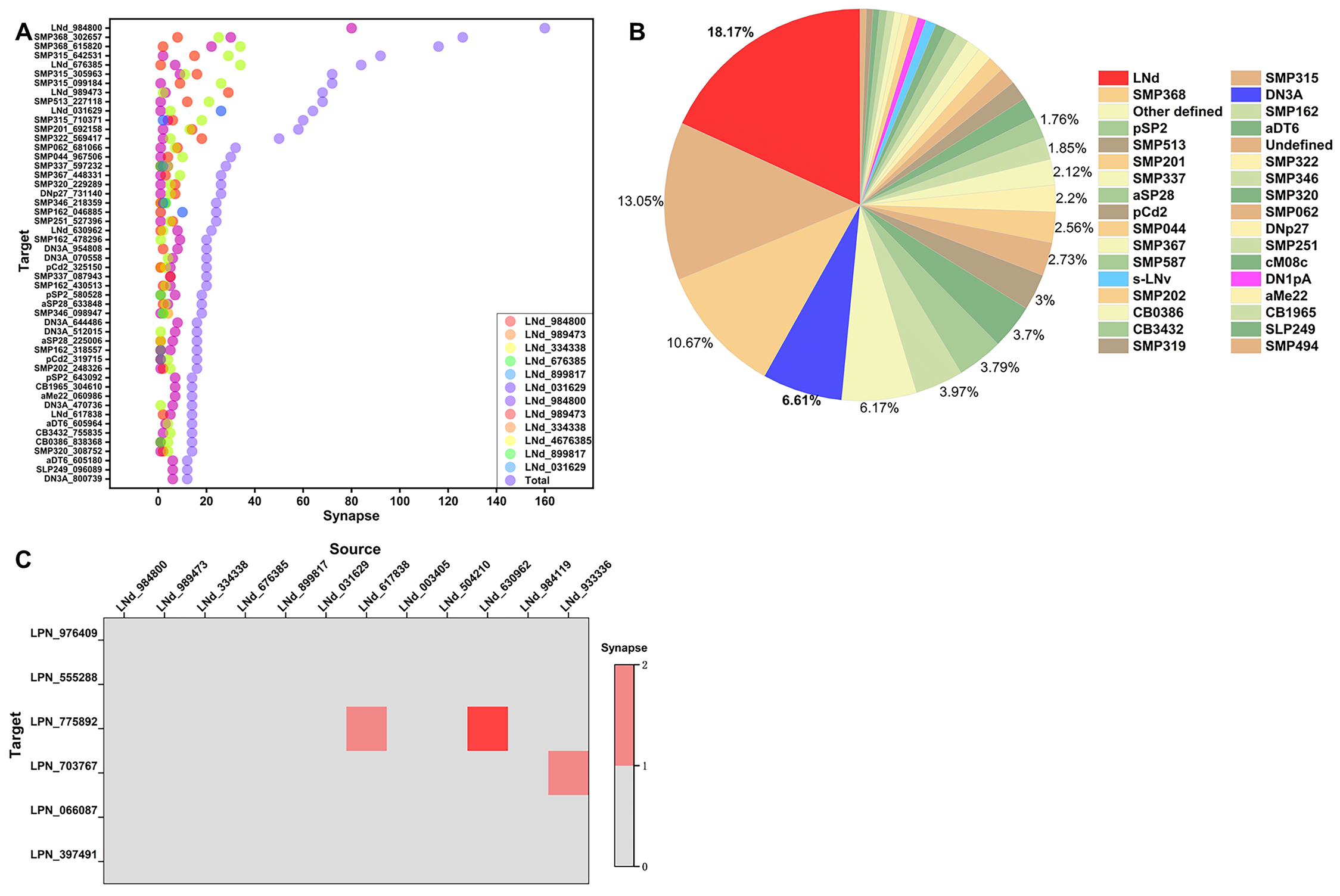

Supplement: S8 Fig — (A) Top 50 postsynaptic neurons of LNds based on FlyWire Data set (v783). (B) Percentage of output from LNds to various neuron types with ≥5 synapses, as predicted using Flywire data set. (C) Synaptic connections from each LNd to each LPN. (TIF) [file pbio.3002918.s011.tif]

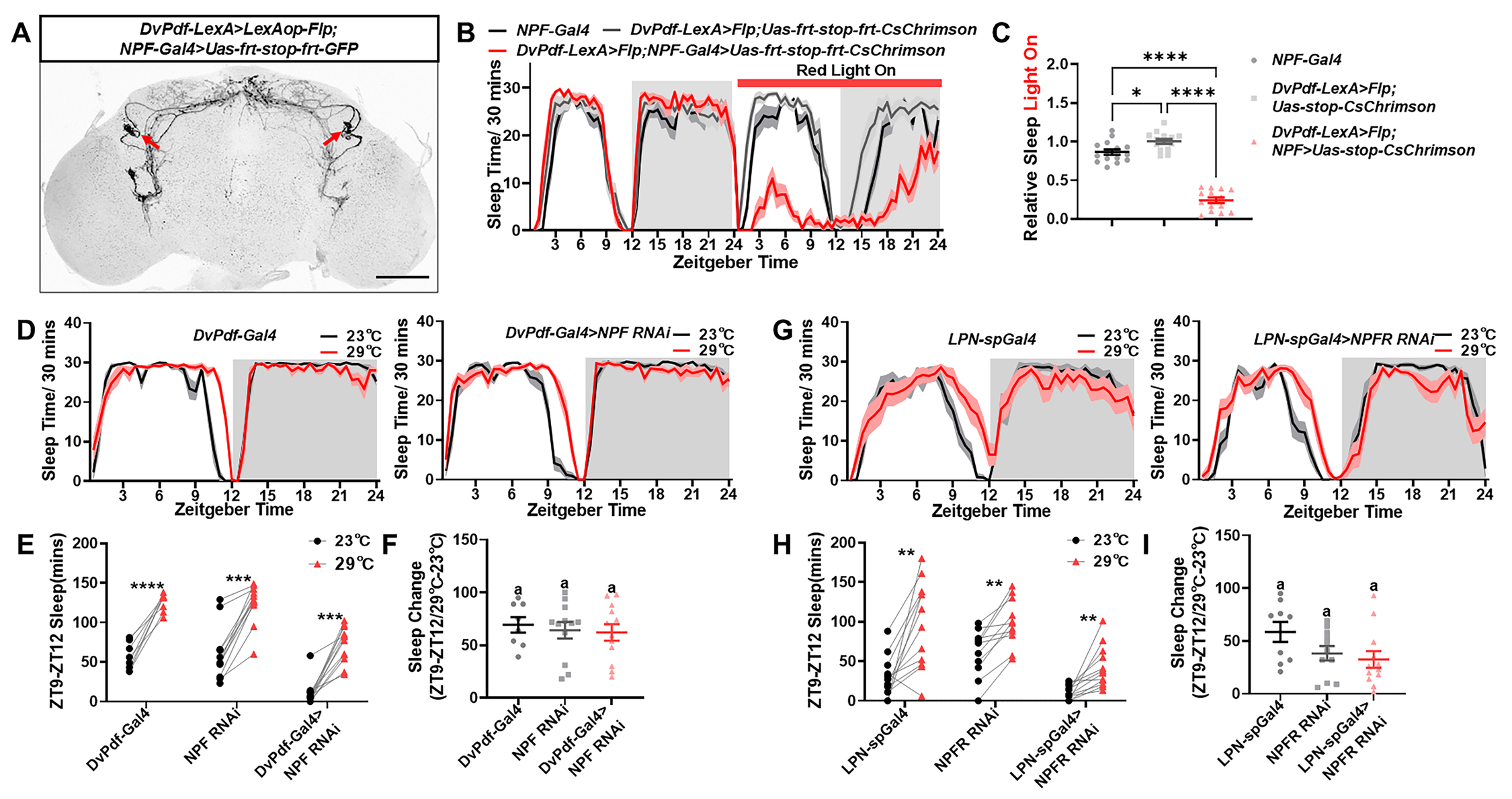

Supplement: S9 Fig — (A) Intersection neurons between DvPdf-LexA and NPF-Gal4 labeled LNds (arrows). Scale bar, 100 μm. (B, C) Optogenetic activation with CsChrimson of the intersectional neurons between DvPdf-LexA and NPF-Gal4 (red lines) causes a strong sleep-inhibiting effect. (D–F) High temperature (29°C) induced evening sleep increase (ZT9-ZT12) was not blocked by NPF knockdown in LNds (DvPdf-Gal4>Uas-NPF RNAi). Quantification of evening sleep (E) and sleep change (F) before and after temperature increase. (G–I) High temperature (29°C) induced evening sleep increase (ZT9-ZT12) was not blocked by NPFR knockdown in LPNs (LPN-spGal4>Uas-NPFR RNAi). Quantification of evening sleep (H) and sleep change (I) before and after temperature increase. Data (C, F, I) were analyzed using Welch’s one-way ANOVA, and same letter a denote no significant difference. Data (E, H) were analyzed with paired t test. *P < 0.05, **P < 0.01, ***P < 0.001, ****P < 0.0001. The raw data in this figure including B, C, D, E, F, G, H, and I can be found in S1 Data. (TIF) [file pbio.3002918.s012.tif]
