# Supplementary material for: Temperature cues are integrated in a flexible circadian neuropeptidergic feedback circuit to remodel sleep-wake patterns in flies
Source: PLoS Biol. 2024 Dec 2;22(12):e3002918. doi: 10.1371/journal.pbio.3002918 (PMC11611155; doi:10.1371/journal.pbio.3002918)
Supplement: Attachment — Submitted filename: The track-changes file.pdf [file pbio.3002918.s019.pdf]

Based on the requirements, following change were made:

1) Title was changed to “**Temperature cues are integrated in a flexible circadian neuropeptidergic feedback circuit to remodel sleep-wake patterns in flies**”.

2) Figure numbering has been updated to continuous alphabetical labels.

3) A raw data file named S1_Data was added to the supplementary material including all mentioned figures.

4) New cites about Flywire dataset were added, mainly in figure legends.

5) The affiliation of the authors have been updated.
